# Basilar Artery Vasospasm as a Cause of Post-Operative Cerebellar Mutism Syndrome

**DOI:** 10.1155/2022/9148100

**Published:** 2022-02-10

**Authors:** Marwa Deghedy, Barry Pizer, Ram Kumar, Conor Mallucci, Shivaram Avula

**Affiliations:** ^1^Department of Oncology, Alder Hey Children's NHS Foundation Trust, Eaton Road, Liverpool L12 2AP, UK; ^2^Department of Neurology, Alder Hey Children's NHS Foundation Trust, Eaton Road, Liverpool L12 2AP, UK; ^3^Department of Neurosurgery, Alder Hey Children's NHS Foundation Trust, Eaton Road, Liverpool L12 2AP, UK; ^4^Department of Radiology, Alder Hey Children's NHS Foundation Trust, Eaton Road, Liverpool L12 2AP, UK

## Abstract

Post-operative cerebellar mutism syndrome (CMS), also known as posterior fossa syndrome (PFS), is a well-recognized and frequent complication of surgery for posterior fossa tumours in children and young people. Its incidence varies between 8 and 31%, and the pathophysiological mechanisms of delayed onset and resolution of cerebellar mutism are not clear, but axonal damage, oedema, and perfusion defects may be involved. Magnetic resonance imaging has failed to reveal a universal anatomical substrate or a single definite mechanism of injury. We present a case of 16-year-old boy who developed CMS three days after resection of a medulloblastoma, a primary fourth ventricular tumour. Early post-operative imaging showed bleeding in the posterior fossa which required evacuation. CT angiography seven days after surgery demonstrated basilar artery vasospasm. Magnetic resonance brain angiography confirmed persistent narrowing of a segment of the basilar artery closely related to a left cerebellopontine (CP) angle peri-operative haematoma. The patient was treated with nimodipine and hypervolemia. The patient started vocalisation without speech five days later with reversal of radiological lesions. Further recovery of post-operative neurological deficits occurred over a protracted period of several months. This case represents a rare cause of post-operative CMS, with rapid initial recovery that occurred after specific treatment directed at the cause. To our knowledge, this is the first reported case showing mutism associated with basilar artery vasospasm with imaging evidence. This case may suggest the need to undertake urgent vascular imaging in selected cases of post-operative CMS.

## 1. Introduction

Post-operative cerebellar mutism syndrome (CMS), also known as posterior fossa syndrome, is a recognized complication of surgical resection of posterior fossa tumours in children [1–3]. CMS is principally characterised by delayed onset mutism and emotional lability or other behavioural changes [1]. We report a unique case of post-operative CMS associated with posterior fossa haemorrhage-associated vasospasm of the posterior circulation, treatment of which improved the vasospasm and associated neurological deficits including mutism.

## 2. Case Report

A 16-year-old boy presented to our institution with a nine month history of ataxia, decreased vision, headache, and vomiting. He was fully conscious with normal speech. Examination showed papilloedema, a right fourth nerve palsy, and an ataxic gait.

**Figure 1:**
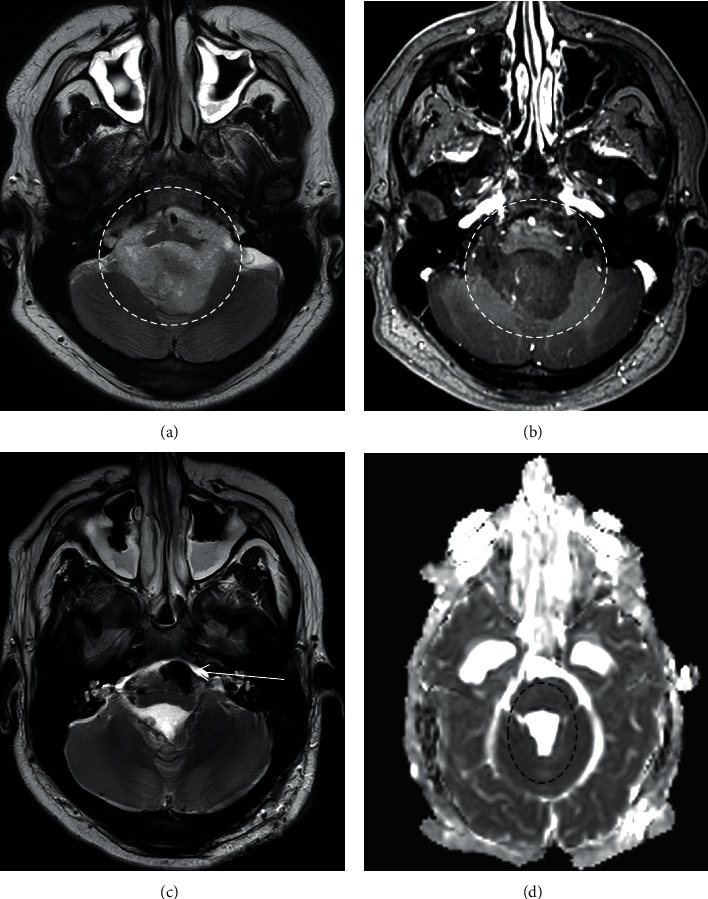
Axial T2 weighted (a) and T1 post-contrast (b) images demonstrating the 4th ventricular tumour (white circle). Axial T2 weighted image (c) following surgery demonstrating susceptibility artefact due to air in the surgical field (white arrow). (d) Complete resection of the tumour with no evidence of diffusion abnormality involving the proximal efferent cerebellar structures on the ADC image (black circle).

MRI of brain and spine showed a obstructive hydrocephalus and a fourth ventricular mass, with maximal diameter of 7.7 cm (Figures 1(a) and 1(b)). There was also a third ventricle metastatic lesion but no evidence of spinal metastasis.

An external ventricular drain was inserted and the tumour was then resected via a combined midline (telovelar approach) and cerebellopontine (CP) angle approach (inverted hockey stick) requiring extensive dissection off the cranial nerves laterally in the CP angle and vertebral artery. The CUSA (Soring) was used for debulk of the deep central parts of the tumour avoiding its use at the interface with cerebellum/peduncles or brainstem.

Intra-operative MRI (ioMRI) confirmed complete resection of the primary tumour with no evidence of diffusion restriction involving the proximal efferent cerebellar pathway (pECP) (Figures 1(c) and 1(d)). Histopathology revealed group 4 medulloblastoma with no MYCN/MYC amplification.

Immediately post-operatively, the boy was able to speak but had difficulty initiating voluntary movement in all limbs. Mutism arose on the third post-operative day. He was agitated and had slow oromotor movement requiring oral suctioning of secretions.

**Figure 2:**
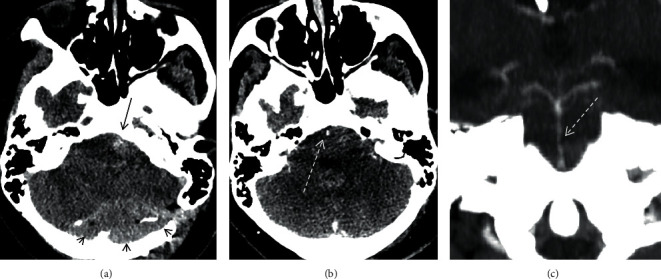
CT scan performed on day 6 following surgery (a) demonstrating extra-axial haemorrhage posterior to the cerebellum (black arrow heads) and haemorrhage in the left CP angle (black arrow). CT angiogram performed following evacuation of the posterior haematoma (b, c) demonstrates irregular narrowing of a large segment of the basilar artery (dotted white arrow). The haematoma in the left CP angle was adjacent to the basilar artery and was not fully encasing it. The basilar artery narrowing extended beyond the haematoma, indicative of vasospasm.

A CT scan on the fourth post-operative day showed an extra-axial haemorrhage, posterior to the cerebellum, with additional fourth ventricle and left cerebellopontine (CP) angle haemorrhage. On follow-up, there was radiological and symptomatic evidence of enlargement of the posterior fossa haemorrhage (Figure 2(a)) leading to surgical evacuation on day 6.

A CT venogram revealed evidence of narrowing involving a segment of basilar artery (Figures 2(b) and 2(c)). The artery was closely related to the left CP angle haematoma and the arterial narrowing extended beyond the haematoma, indicative of an element of vasospasm. On retrospective review, this segment of the basilar artery was narrow on the ioMRI and very closely related to the haematoma that was indistinct (almost isointense to CSF). Therapy was given with enteric nimodipine (60 mg every 4 hours) together with hypervolemia (>3 L/day) for 14 days, components of the so-called Triple H therapy (induced hypertension, hypervolemia, and haemodilution) widely used to prevent and treat cerebral vasospasm, e.g., after aneurysmal subarachnoid haemorrhage [4].

He remained neurologically stable throughout, alert and obeying commands although mute, weak, and ataxic needing assistance to sit and transfer. On the twelfth post-operative day, the patient started tuneful vocalisation.

**Figure 3:**
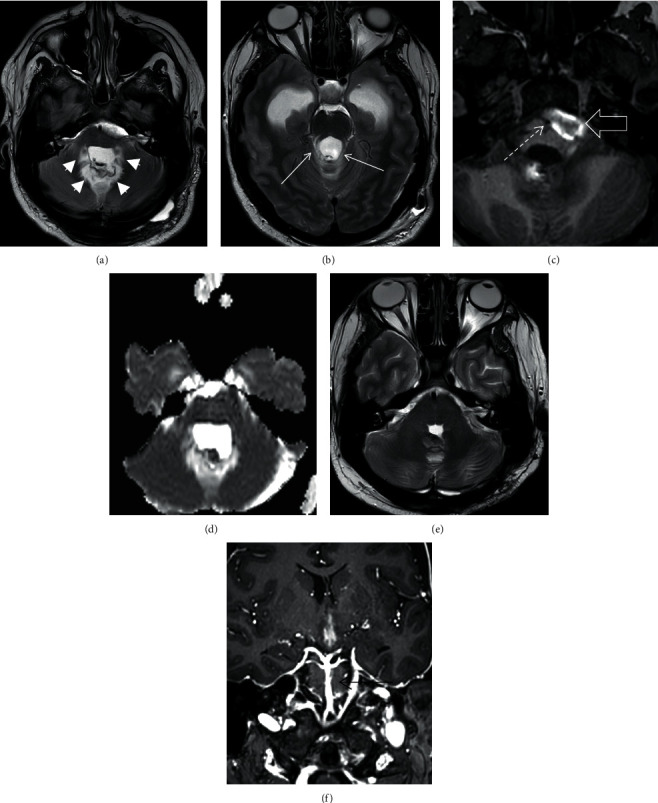
Axial T2 weighted images (a, b) from the MRI performed on day 14 demonstrate bilateral hyperintensity in the regions of the dentate nuclei, middle cerebellar peduncles (white arrow heads), and the superior cerebellar peduncles (white arrows). The T1 weighted image (c) demonstrates the narrow basilar artery (white dashed arrow) and the adjacent haematoma (white open arrow). The ADC image (d) demonstrates unrestricted diffusion in the region of the T2 abnormalities. MRI scan performed 6 weeks following surgery demonstrates complete resolution of the T2 abnormality (e) and normal calibre of the basilar artery (black arrow) on the post-contrast image (f) as compared to the changes seen on Figure 2(c).

Fourteen days from first surgery, a ventriculoperitoneal shunt was placed for dilated ventricles. MR angiography demonstrated persistent narrowing of the inferior segment of the basilar artery closely related to the left CP angle haematoma. There was significant T2 hyperintensity involving bilateral dentate nuclei, middle cerebellar peduncles, and superior cerebellar peduncles. These areas demonstrated unrestricted diffusion (Figures 3(a)–3(d)).

On the sixteenth post-operative day, the patient started speaking using single words and gradually regained speech in sentences.

MRI brain six weeks following the primary resection demonstrated normal calibre of the basilar artery with complete resolution of the cerebellar T2 signal and diffusion abnormalities and the left CP angle haematoma (Figures 3(e) and 3(f)).

Adjuvant chemotherapy (POG 9031) and risk-adapted craniospinal radiotherapy including a boost to the sites of primary and metastatic disease were administered. The third ventricular metastatic mass was partially resected prior to radiotherapy. He completed the planned courses of chemotherapy (3 courses cisplatin and etoposide and seven courses of cyclophosphamide and vincristine).

Our patient showed gradual improvement of speech to produce descriptive sentences at 2 months post-surgery. At last review, 25 months since surgery, he has completed the 7 planned courses of consolidation chemotherapy (cyclophosphamide and vincristine). He has shown remarkable improvement in speech and is now communicating with short sentences. Recent video fluoroscopic swallow was normal, and he is walking without support.

## 3. Discussion

The case described here presented with features compatible with the diagnosis of post-operative CMS as defined by the Posterior Fossa Society [1]. Our case is unique in the development of post-operative CMS following basilar artery vasospasm, clearly demonstrated in the intra-operative and post-operative scan. This report supports the proposition of vasospasm as a possible causative mechanism of CMS due to the delayed onset and the transient nature of both phenomena [5–11].

Vasospasm is a recognized phenomenon associated with neurosurgery. In the setting of tumour resection, vasospasm can be induced by pre-operative narrowing or encasement of the tumour, imbalance of vascular tone, subarachnoid haemorrhage, and spillage of tumour in the subarachnoid space [12]. Whilst sellar and middle cranial fossa tumour surgery has demonstrated localised vasospasm, posterior fossa tumour resection tends to result in diffuse vasospasm causing extensive neurological deficits including cortical blindness, hemiparesis, and cranial nerve paresis [13–16]. The lack of dedicated studies, including imaging evidence, has been a limiting factor in support of the hypothesis that cerebellar perfusion deficits secondary to arterial vasospasm cause CMS [11].

Our patient also developed atypical cerebellar deep white matter abnormality, resolving by six weeks along with resolution of basilar artery vasospasm, concordant with improvement of speech and other neurological deficits.

The nature of the white matter abnormality is unknown. It does not conform to a specific arterial territory and did not demonstrate diffusion restriction at the time of the scan. The cerebellar deep white matter is the border zone between the penetrating branches of the posterior inferior cerebellar artery (PICA), superior cerebellar artery (SCA), and the anterior inferior cerebellar artery (AICA), and infarcts in this area have been thought to represent watershed infarcts [17]. In our extensive experience, we have never noted this pattern of abnormality in the post-operative period in children with CMS. Intra-operative and post-operative imaging also did not show direct injury to the pECP structures, a finding that has been observed with the CMS cohort in our institution. In a recent study, diffusion restriction around the surgical cavity was significantly associated with CMS [18]. In our case, however, the cerebellar white matter demonstrated unrestricted diffusion. We speculate that the basilar artery vasospasm seen uniquely in this case may have caused reversible injury of the cerebellar deep white matter including the structures of the pECP. The pECP includes the dentate nucleus and the superior cerebellar peduncles, structures that have been linked to the development of CMS [8].

The impact of induced hypertension and hypervolemia in this case is unclear although the authors would suggest consideration of such treatment in similar cases. With respect to treatment of CMS, a number of drugs have been reported as showing promise although none have clear efficacy, and further studies are clearly required [19]. The role of steroids in prevention and treatment is unclear.

Our case highlights the important role of MR angiography sequence for identifying vasospasm in children with mutism after cerebellar tumour surgery, particularly if haemorrhage close to the vertebrobasilar artery is detected on peri-operative imaging.

## Data Availability

The data supporting the results of this case report are available from the corresponding author upon request.
